# Robust whole-hand spatial manipulation via energy maps with caging, rolling, and sliding

**DOI:** 10.3389/frobt.2023.1281188

**Published:** 2023-11-22

**Authors:** Walter G. Bircher, Andrew S. Morgan, Aaron M. Dollar

**Affiliations:** GRAB Lab, Mechanical Engineering and Materials Science, Yale University, New Haven, CT, United States

**Keywords:** spatial manipulation, dexterous manipulation, whole-hand manipulation, potential energy, caging, design

## Abstract

Humans regularly use all inner surfaces of the hand during manipulation, whereas traditional formulations for robots tend to use only the tips of their fingers, limiting overall dexterity. In this paper, we explore the use of the whole hand during spatial robotic dexterous within-hand manipulation. We present a novel four-fingered robotic hand called the Model B, which is designed and controlled using a straight-forward potential energy-based motion model that is based on the hand configuration and applied actuator torques. In this way the hand-object system is driven to a new desired configuration, often through sliding and rolling between the object and hand, and with the fingers “caging” the object to prevent ejection. This paper presents the first ever application of the energy model in three dimensions, which was used to compare the theoretical manipulability of popular robotic hands, which then inspired the design of the Model B. We experimentally validate the hand’s performance with extensive benchtop experimentation with test objects and real world objects, as well as on a robotic arm, and demonstrate complex spatial caging manipulation on a variety of objects in all six object dimensions (three translation and three rotation) using all inner surfaces of the fingers and the palm.

## 1 Introduction

When all surfaces of a robotic hand are used for manipulation, rather than just the fingertips, its dexterity can be markedly increased (Vassura and Bicchi, 1993). It is this principle upon which our work is based. This observation has been made by other researchers within the field of robotic manipulation in the past and can be easily understood simply by watching humans manipulate. While many traditional models of manipulation are built around an assumption of fixed fingertip contact between the hand and the object (Salisbury and Roth, 1983; Kerr and Roth, 1986), this work allows contacts to be established and broken freely along all inner surfaces of the hand. This is possible because of the energy-based forward motion model at the heart of this work which does not require precise modeling of contact mode, friction, or contact forces. This is in contrast to previous work in the literature (Brock, 1988; Trinkle, 1989; Howe and Cutkosky, 1996), which shows that accurate prediction of contact motion depends on precisely measuring contact force magnitude and direction, as well as coefficient of friction which is known to change over time (Yu et al., 2016), and local surface geometry (Okada, 1982; Cole et al., 1988; Montana, 1988). This is a tradeoff—namely the model is based on many simplifying assumptions—but as shown in this work and in previous work (Bircher et al., 2021), these assumptions lead to favorable results in many common manipulation scenarios.

This work shows how the potential energy-based motion model from (Bircher et al., 2021) can be formulated for spatial manipulation in three dimensions, and how it can be used to design spatial manipulators. The work begins in simulation and culminates in the development and testing of a novel spatial manipulator called the Model B. This hand has four fingers, each consisting of fully actuated two link serial chains; two opposing fingers on prismatic bases and two on a rotary base for abduction about an axis orthogonal to the palm. The hand was designed to perform manipulation while maintaining a cage on the object (Makita and Maeda, 2008a; Diankov et al., 2008; Rodriguez et al., 2012), allowing contact constraints to be relaxed and helping to enable more adventurous manipulation primitives without increasing the risk of object ejection.

Many robotic hands have been designed specifically to achieve some sort of manipulation or grasping task. The utility of certain design features, such as underactuation compliance, has long been embraced for graspers, as it enables easy and robust open-loop grasping of novel object geometries (Dollar and Howe, 2010). Researchers have often taken these simple underactuated hands a step further than grasping, showing that they can be used for simple open-loop manipulation tasks as well (Odhner et al., 2013; Ma et al., 2017). It has been shown that with subtle design changes, these simple underactuated hands can have their manipulation capability greatly increased for certain tasks, such as planar rotation of an object (Bircher et al., 2017; Ma et al., 2023). Other hands have been designed to achieve large amounts of object translation (Kakogawa et al., 2016) within the hand, or even both rotation and translation (Tincani et al., 2012; McCann and Dollar, 2017).

There are some hands that have been designed specifically for planar whole-hand manipulation, and even fewer for whole-hand spatial manipulation. Those that have been designed for whole-hand manipulation often have tens of degrees of freedom (DOF) and need complex controllers to perform manipulation primitives (ShadowRobot, 2005; AndrychowiczOpenAIMarcin et al., 2020). This illustrates the classic tradeoff between added DOF and increased controller complexity. This work shows how, with careful design, useful dexterity can be achieved without an excessive number of actuators, without necessitating overly complex control or expensive sensors. In the literature there are many examples of controllers ranging in complexity from pure kinematics (Maeda and Asamura, 2016; Sundaralingam and Hermans, 2017), to more detailed controllers with contact modelling of rolling and sliding (Trinkle et al., 1993), to those that include full blown dynamics modelling (Li et al., 1989).

Those controllers that include contact modelling typically implement force-closure conditions to ensure that an object is not dropped during manipulation. In this work however, the Model B was designed to maintain a loose cage on an object while manipulating. Future more optimized hand designs could very well utilize existing models of spatial caging (Makita and Maeda, 2008b) during the design process to guarantee a cage over a wide range of object shapes and sizes.

**FIGURE 1:**
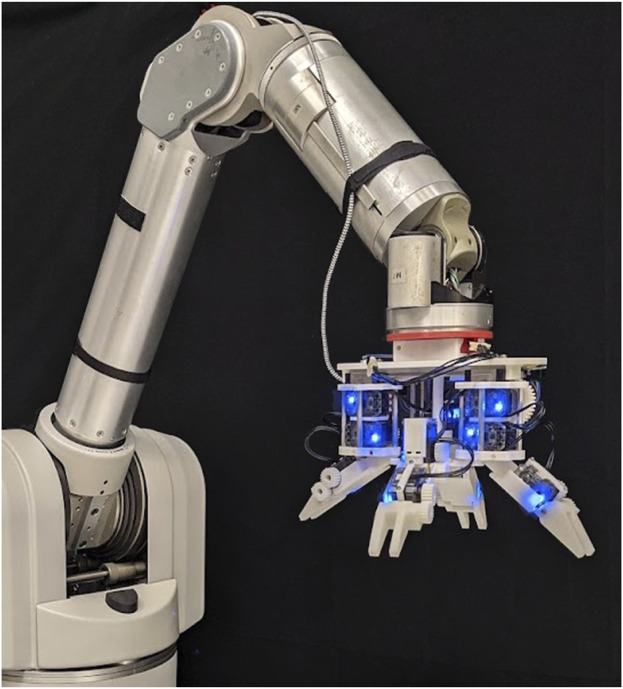
The Model B is a four fingered hand designed for spatial within-hand caging manipulation.

The contributions of this work include the formulation of the potential energy-based forward motion model (Ma et al., 2017; Bircher et al., 2019; Ma et al., 2019; Bircher et al., 2021) from 2D to 3D, the formulation of the manipulation metric from (Bircher et al., 2021) in 3D, and the development and demonstration of a new hand, the Model B shown in Figure 1. The rest of this work is organized as follows. In Section II we detail the energy model formulation and the manipulation metric formulation. In Section III we present the robotic hands that were assessed using the metric. In Section IV the simulation results are presented. Section V lays out the design of the Model B, and Section VI showcases the experimental results from the hand with both benchtop and robot arm testing. Finally, Section VII includes detailed discussion of the hand and its implications for manipulation research.

## 2 Energy-based forward motion model

**FIGURE 2:**
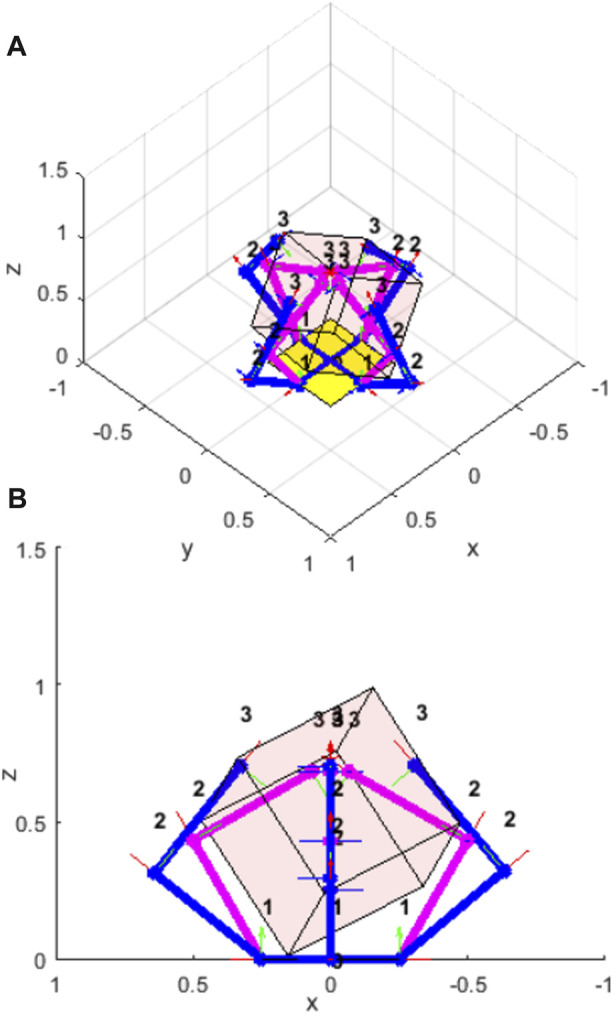
A four-fingered hand in simulation with a cube. The pink links show the commanded finger positions, the blue links show the displaced finger positions due to the geometry of the cube. The potential energy of the system is due to the difference in the commanded and displaced joint positions. **(A)** isometric view; **(B)** side view.

The manipulation model described in this work extends previous work done on the design of a dexterous hand for planar caging manipulation to three dimensions. This theory is based on the idea that with enough mechanical work, a position controlled motor can be back driven past its set point, gaining potential energy during the process. It was originally inspired by the work of energy-based analysis of grasping in (Krag et al., 2010; Mason et al., 2012). All nomenclature used in this section is defined in Table 1. The potential energy of this motor gained during a displacement is

$$U={-\tau }_{m}\left(\theta_{d}-\theta_{sp}\right)$$ (1)

where $\tau_{m}$ is the torque supplied by the motor, $\theta_{sp}$ is the commanded set point of the motor, and $\theta_{d}$ is the new position of the shaft after being displaced. A displacement can occur due to an external disturbance that overcomes the torque of the motor. Such a system may include a transmission, which can be represented by K , which simply relates the amount of joint displacement to the actual displacement felt by the motor. In a system with multiple actuators, the overall potential energy gained by their combined displacements can be represented by

$$U=\sum_{i=1}^{n}\tau_{i}K_{i}\left(\alpha_{i}-\theta_{i}\right)$$ (2)

where there are n actuators in the system. As an example, a two-link finger can be displaced at the fingertip by contacting an immovable object, displacing the motors at each of its joints. In this scenario, we can then calculate the potential energy gained by the motors during this displacement. This is illustrated in Figure 2.

**TABLE 1:** Nomenclature.

𝑈	Potential energy
𝜏	Torque generated by motor
$\theta_{d}$	Displaced joint position
$\theta_{sp}$	Motor set point
𝑛	Number of actuators
𝐾	Transmission ratio
𝑅	Rotation matrix
𝑢	Joint position vector in Cartesian space
𝑃	Set of Cartesian positions of all finger joints
$T_{ab}$	Transformation matrix from frame 𝑎 to 𝑏
𝑁	Dimension of system, 3 for spatial
𝑆	Set of all object boundary points
𝑠	Object boundary point in Cartesian space
𝑈∗	Minimum energy
𝑞	Object pose
U	Minimum energy-valued scalar field
𝑄	Number of distinct object poses
𝛾	Vector field
𝑤	Wrench vector
𝑥,𝑦,𝛽	Planar object pose coordinates
Θ	Number of distinct actuation inputs
𝑊	Set of vectors at object pose for all 𝜃𝑠𝑝
$M_{avg}$	Hand caging manipulability score
R,P	Abbrs. for revolute and prismatic joints

### 2.1 System kinematics

Using forward kinematics, the displacement of a motor or a joint can easily be mapped to a corresponding Cartesian displacement of a robot link. Specifically, we can use transformation matrices constructed from Denavit-Hartenberg parameters to relate these parameters. The Cartesian position of a frame affixed to joint k of a serial chain linkage with n links in a fixed global frame can be extracted from the transformation matrix Tk0 as $u_{k}$ , where

Tk0=Rkuk001=∏i=1kTii−1θi,k≤n uk0∈RN (3)

such that Tii−1θi is the homogeneous transformation matrix that transforms points from link i−1 to link i , uk0 is the joint position vector for the k th joint, and $R_{k}$ is the rotation matrix associated with the k th joint. In this work we assume that all standard Denavit-Hartenberg conventions apply, and also that a joint cannot hyperextend past the previous link. In other words, the robotic fingers described in this work can curl inwards on one extreme, and form a straight serial chain on the other. For each finger, let the Cartesian positions of each joint be Contained in the set $P_{\theta_{sp}}$ at the commanded actuation input $\theta_{sp}$ such that

Pθsp=uθsp,kk−1|k=1,…,n,ukk−1∈R3 (4)

Let there be an object to be manipulated described by a set S of m boundary points with respect to a joint-affixed frame i such that

Sqi=sq,iji|i=1…n,j=1,…,m,sji∈R3 (5)

where sji∈R3 for spatial objects. The challenge of this work is to determine the displacement at the joint or motor level caused by the hypothetical forcible placement of an immovable object, such that it displaces the hand. In other words, given a known object pose and hand configuration, how is the hand displaced assuming the object cannot be moved? And furthermore, what is the associated potential energy gained by the hand due to this displacement? These questions are answered by formulating the problem as an optimization program wherein the overall system energy is minimized subject to kinematic and non-penetration constraints that keep the fingers from passing through the object. This problem is challenging because we do not know exactly where the fingers will contact the object, and contact anywhere along the surfaces of the fingers or palm is feasible—unlike more traditional models of robotic manipulation which assume object contact only occurs at the fingertips.

### 2.2 Energy minimization

Given an external displacement, the hand will reconfigure to its lowest energy configuration. Thus, we formulate an optimization program to minimize the system’s total potential energy subject to constraints. The optimization program results in the scalar minimal system energy value as well as the associated minimal energy configurations of all joints of hand. It is formulated as follows

$$U_{\theta_{sp},q}^{*}=\min_{\theta_{d}}\sum_{i=1}^{n}\tau_{i}K_{i}\left(\theta_{d_{i}}-\theta_{sp_{i}}\right)$$ (6)

$$s.t.\;0\leq \theta_{d_{i}}\leq \theta_{sp}\;\forall i$$

$$f\left(x\right)\leq 0$$

where $U_{\theta_{sp},q}^{*}$ is the minimum system energy given commanded actuation input $\theta_{sp}$ and object pose q . The optimal solution is a vector $\theta_{d}^{*}$ consisting of the displaced joint positions. The constraint $f\left(x\right)\leq 0$ in this optimization program represents a generalized non-penetration or collision constraint that prevents the fingers from penetrating the object, and $0\leq \theta_{d_{i}}\leq \theta_{sp}$ prevents the joints from exceeding their travel limits (due to mechanical hard-stops at each joint). The collision constraint can be implemented in any number of ways, but it is advantageous to find a method that is computationally efficient since it will need to be calculated many times during minimization. The resulting joint positions must also be less than the commanded set points, since the object cannot pull the finger past where it is actuated. For a hand with multiple fingers, we can simply solve the optimization program once for each finger and sum the resultant energies to obtain the overall potential energy of the hand at each configuration of the system.

### 2.3 Energy fields

Solving the energy minimization for all feasible poses of the object where manipulation is possible yields the energy field for a given system configuration and actuation input, represented by

$$U_{\theta_{sp}}=\left\{U_{\theta_{sp},q}^{*}|q=1,\ldots ,Q\right\}$$ (7)

where $U_{\theta_{sp}}$ is a scalar field that shows how an object will move when manipulated. Namely, it enables visualization of the system’s potential energy contours, including the workspace region containing the lowest energy, which is where the object will be most likely to settle once the given actuation has been applied to the hand.

### 2.4 Gradient of energy map

**FIGURE 3:**
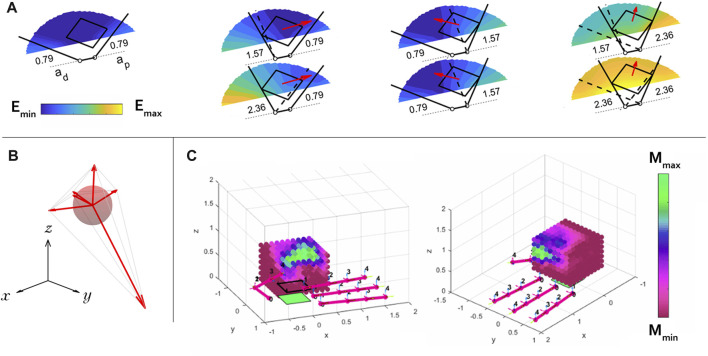
**(A)** Seven energy maps are shown for seven actuation input combinations for a simple planar hand with two revolute fingers manipulating a square object in the plane. The commanded position (actuation input) to each finger is written in radians next to its joint. This hand is used as an example since it is easier to visualize a planar system’s energy map than that of a spatial system. The gradient of each energy map is calculated locally at the object’s drawn location, and is represented by a red vector. These vectors show how an object will move given its current location with the written actuation input to the system. **(B)** We find the largest ball inscribed inside the convex hull formed by the tips of the gradient vectors, which is a measure of manipulability of a hand-object system in a specific configuration. The example shown here is only three-dimensional for the purposes of illustration, but in this work we are actually finding 6-dimensional hyperspheres inscribed in the object’s spatial configuration space. **(C)** The manipulability of the Allegro Hand with a cube is calculated over a grid and shaded accordingly. The model predicts that the hand is only capable of achieving high levels of dexterity (green) where the thumb and index fingers meet.

The energy field gradients result in vector fields, lending even more intuition about the motion of an object given a system configuration and actuation input (see Figure 3A). Specifically, the vector field consists of net wrench vectors that will be applied to the object under the given assumptions. This vector field can be written as

$$\gamma_{\theta_{sp}}=-\nabla_{x,y,\beta }U_{\theta_{sp}}$$ (8)

for each scalar field $U_{\theta_{sp}}$ with actuation input $\theta_{sp}$ . The vectors comprising the field $\gamma_{\theta_{sp}}$ are concretely wrench vectors $w_{q}\in R^{6}$ are of the form $w_{q}=\left[{f_{x}}_{q},{f_{y}}_{q},{f_{z}}_{q},{\tau_{x}}_{q},{\tau_{y}}_{q},\tau_{z_{q}}\right]$ and each could be potentially realized by the object—potentially because the model does not take friction into account, ideal object and hand geometry are assumed, and actuation is assumed to be ideal. Thus, the actuation that produces these wrenches is a necessary, rather than a sufficient condition for the physical existence of these wrenches. The set of all vector fields $\Gamma =\left\{\gamma_{1},\ldots ,\gamma_{\Theta }\right\}$ calculated over the set Θ of all possible actuator inputs is useful for evaluating the overall manipulation capabilities of a hand. For a given system pose, the set of all possible vectors corresponding to all possible actuation inputs can be written as $W_{q}=\left\{w_{q,1},\ldots ,w_{q,\Theta }\right\}$ where the span of these vectors represent all possible wrenches that could be applied to the object in its current configuration.

### 2.5 Manipulation metric

The convex hull of the set $W_{q}$ of all possible vectors that can be applied to the object in a given configuration is $Conv\left(W_{q}\right)$ . The radius of the largest ball that can be inscribed inside this hull represents the largest wrench that can be imparted to the object in any direction (see Figure 3B). This is a useful metric for judging the hand’s manipulability. Considering the average, minimum, or maximum radius over the hand’s whole workspace tells us useful information about its overall manipulability. This can be used to compare one hand design to another. In this work, we consider the average radius of the largest ball over the hand’s entire workspace, $M_{avg}$ . The algorithm used to perform this calculation is detailed in our previous work (Bircher et al., 2021) and in the literature (Ziegler, 2012; Boyd and Vandenberghe, 2023). The larger the inscribed ball, the larger the wrench that can be exerted on an object in any direction. The radius of this ball (or n-sphere) is calculated by first finding the polytope representation of $Conv\left(W_{q}\right)$ , which is defined by the intersection of hyperplanes. Recall that any polytope is defined by the intersection of hyperplanes $\langle h_{i},w\rangle \leq b_{i}$ . Given the hyperplane representation, the maximum radius can be computed with the quadratic program

$$w_{q}^{*}=\underset{w}{\max}{\left\|w\right\|}^{2}$$ (9)

$$s.t.\;\left\langle h_{1},,,w\right\rangle \leq b_{1}$$

$$\left\langle h_{2},,,w\right\rangle \leq b_{2}$$

⋮

$$\left\langle h_{n},,,w\right\rangle \leq b_{n}.$$

In this work, the ball lives in a six dimensional space (the spatial wrench space). The score for a hand $M_{avg}$ is the average radius over entire workspace, averaged over all objects (see Figure 3C).

## 3 Simulation of robotic hands

Eleven commercially available, open-source, or novel robotic hand topologies were simulated in this work and their manipulation capabilities were quantified based on the metric described in the previous section. The goal of this work was not to find a globally-optimal design for spatial manipulation in a strict sense, but simply to explore the space of existing designs and implement a configuration that has good enough performance to demonstrate the feasibility of spatial manipulation of this type. (Previous work optimized over a wide range of planar designs (Bircher et al., 2021), and the parameter space is orders of magnitude larger for spatial configurations).

The simulation of these hands demonstrates that the energy-based motion model can be used with hands consisting of many actuators, such as the Allegro hand (16 actuators). The theoretical manipulability of each hand was simulated on the Yale High Performance Computing resource, taking anywhere from less than a minute to several days depending on the complexity of the hand.

**FIGURE 4:**
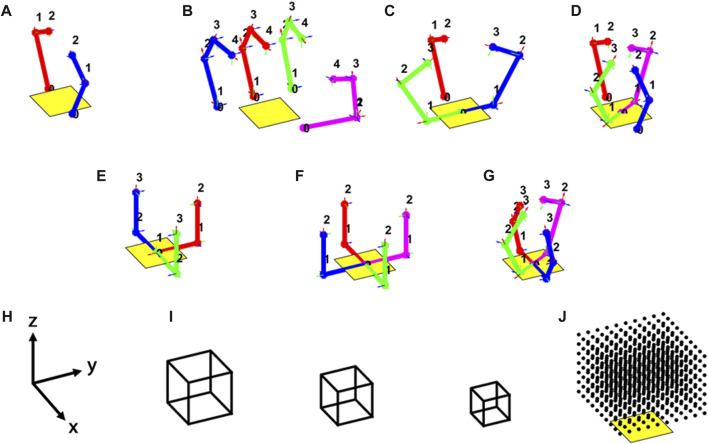
Eleven hands were simulated to manipulate cubes in this work throughout their workspace. The kinematic topologies for are shown for the following simulated hands: **(A)** underactuated and fully actuated T42; **(B)** Allegro Hand; **(C)** underactuated and fully actuated Model O; **(D)** underactuated and fully actuated Model Q; **(E)** H1; **(F)** H2; **(G)** Model **(B,H)** simulation world frame; **(I)** simulated cubes; **(J)** workspace grid (it is centered with the palm and raised one-half cube side length along the *z*-axis).

To begin, five existing hand designs were simulated, as shown in Figure 4. These include commercially available hands such as the Allegro hand, as well as open-source hands such as the Yale OpenHand Model T42, Model Q, and Model O (Odhner et al., 2014a; Odhner et al., 2014b; Ma and Dollar, 2014; Ma and Dollar, 2017). We found that simulating these hands, especially the OpenHand models, was very valuable in establishing intuition about the overall performance in known terms, as there are many examples of these hands manipulating various objects within the literature. Six novel hand designs were also simulated. These include fully actuated versions of the Yale OpenHand Model T42, Model Q, and Model O, as well as completely new hand topologies. In this work we refer to the new topologies as the H1, the H2, and the Model B. The Denavit-Hartenberg parameters used to simulate all of these hands are shown in Table 2. In the case of underactuated hands, the joint coupling is also noted.

**TABLE 2:** Parameters for simulated hands.

		Finger	Joint	DH parameters				
				Type	a	-	-	-
Hand	T42	1,2	1	R	0.63	0	0	0
			2	R	0.37	0	0	0
	Model O	1,2	1	R	0.403	𝜋/2	0	0
			2	R	0.63	0	0	0
			3	R	0.37	0	0	0
		3	1	R	0.63	0	0	0
			2	R	0.37	0	0	0
	Model Q	1,2	1	R	0.6	0	0	0
			2	R	0.4	0	0	0
		3,4	1	R	0.25	𝜋/2	0	0
			2	R	0.6	0	0	0
			3	R	0.4	0	0	0
	H1	1,2	1	R	0	𝜋/2	0	0
			2	P	0	𝜋/2	0.5	−𝜋/2
			3	R	0.5	0	0	0
		3	1	P	0	𝜋/2	0.5	0
			2	R	0.5	0	0	0
	H2	1–4	1	P	0	𝜋/2	0.5	0
			2	R	0.5	0	0	0
	Model B	1,2	1	P	0	𝜋/2	0.33	0
			2	R	0.33	0	0	0
			3	R	0.33	0	0	0
		3,4	1	R	0.25	𝜋/2	0	0
			2	R	0.6	0	0	0
			3	R	0.4	0	0	0
	Allegro	1–3	1	R	0	𝜋/2	0	0
			2	R	0.54	0	0	0
			3	R	0.384	0	0	0
			4	R	0.387	0	0	0
		4	1	R	0.621	0	0	0
			2	R	0	−𝜋/2	0	0
			3	R	0.514	0	0	0
			4	R	0.387	0	0	0

## 4 Simulation results

**FIGURE 5:**
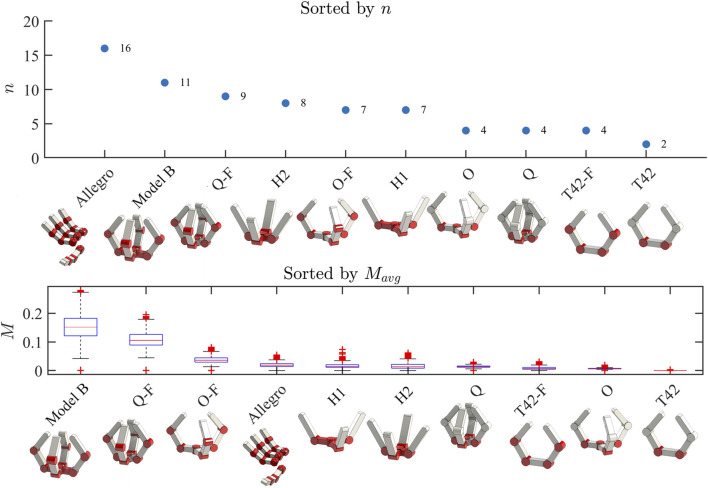
Top Panel: the hands simulated in this work are shown and sorted by the number of actuators. An ‘F’ in a hand’s name indicates that it is the fully actuated version of an underactuated hand from Yale OpenHand. Bottom Panel: The same hands are now sorted by their performance based on the manipulation metric $M_{avg}$ described in section II.F.

The average manipulability was calculated for each hand according to them metric in Eq. 9 and the results are shown in Figure 5. The results show that in general, hands with more actuators perform better—but not exclusively so. Some hands with many motors, such as the Allegro hand, do not perform very well, likely do to the kinematic redundancy of its fingers. After all, the Allegro Hand was likely designed for fingertip manipulation, rather than whole-hand manipulation as is being assessed in this work. It seems that in order to achieve higher dexterity throughout the workspace in a whole-hand sense, it is advantageous to use sets of opposing fingers.

Several hands including the underactuated and fully actuated versions of the T42 and the underactuated version of the Model O had virtually negligible spatial manipulation capability. This matches nicely with intuition, as the T42 topologies can clearly only manipulate in the plane of the fingers, and have no ability to control the object orthogonal to that plane. This means that in theory, manipulation capability should be zero according to the metric. The underactuated Model O suffers from a similar problem, although it does have the ability to theoretically manipulate in more directions. In its case, the low manipulability instead comes from its underactuation, rather than its kinematic topology. It was found that the Model B had the highest manipulation capabilities of any of the simulated designs. This is interesting given that it does not have the highest number of actuators—as intuitively we expected manipulability to scale with number of actuators. This shows that (perhaps unsurprisingly) kinematic topology has a large effect on the overall dexterity of a hand, and that it is not simply enough to have lots of motors—they must be arranged in a clever way to be useful. The Model B performed over three times better than all hands except for the fully actuated Model Q (Q-F), which performed about two-thirds as well. We hypothesize that the reason for the Model B’s stellar theoretical performance is in large part due to its ability to perform a power to pinch transition with its prismatic finger pair. None of the other hands can so easily push objects out away from the palm, simply because their kinematic topologies are not set up to do so.

## 5 The model B

**FIGURE 6:**
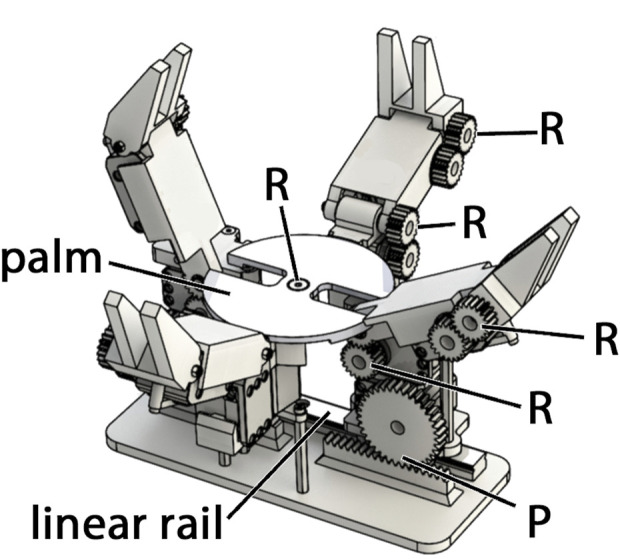
A rendering of the Model B hand. It is comprised of four fingers—two pairs of identical opposing fingers. One set is prismatic, the other rotates about the axis of the palm. Each finger has a proximal and distal link. The distal links of each finger interdigitate with the opposing finger.

A physical hand was designed based on the simulated design parameters for the Model B (see Table 2) and it is shown as a CAD rendering in Figure 6. The hand has eleven inexpensive smart servos, specifically Dynamixel XL-320s. It has an 80 mm diameter palm, two opposing prismatic fingers with 72 mm proximal links and 46 mm distal links. It also has a pair of coupled abduction fingers with 30 degrees of rotation about the center axis of the palm. Each abduction finger has a 57 mm proximal link and a 46 mm distal link. All parts, including gears and rack, were 3D printed out of ABS using a Stratasys uPrint. Revolute joints were realized using ball bearings and shoulder bolts, and the prismatic joints were supported using off the shelf carriages and rails, and actuated by rack and pinion.

Bench top experiments were performed to assess the hand’s ability to manipulate a variety of objects in a controlled environment. Open loop manipulation primitives were manually determined and hard coded such that manipulation motions could be chained together. These manipulation primitives include left-right shift motions, power to pinch motions, pinch to power transitions, roll motions, and yaw motions—both in clockwise and counter clockwise directions, both right side up and upside down.

The hand’s robust manipulation ability was demonstrated by performing repeated manipulation primitives both in the bench top setting and on a 7 degrees of freedom (DOF) Barrett Whole Arm Manipulator (WAM) robotic arm. These demonstrations were performed using the open loop motion primitives described in the previous section. A variety of objects were manipulated including some from the Yale-CMU-Berkeley (YCB) Object Set (Calli et al., 2015), as well as painted wooden cubes and a soft knitted cube.

**FIGURE 7:**
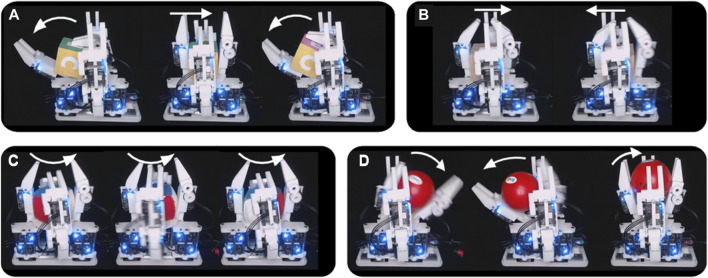
The Model B can manipulate objects with gravity into the palm. **(A)** The hand performs a “yaw” motion with the wooden cube; **(B)** The hand performs a left-right shift with a wooden cube; **(C)** The hand performs a “roll” motion with the knitted cube; **(D)** The hand performs a power to pinch transition against gravity with a ball.

**FIGURE 8:**
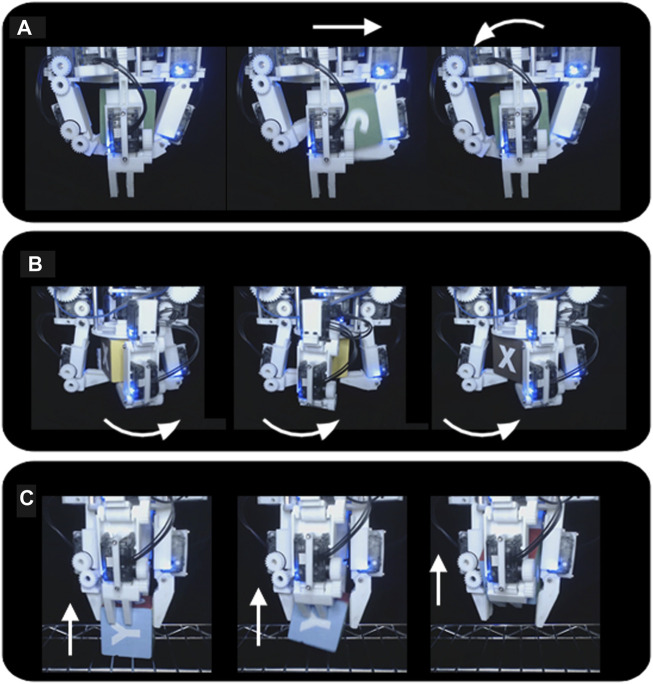
The Model B can manipulate a wooden cube against gravity. **(A)** The hand performs a “yaw” motion with the cube; **(B)** The hand performs a “roll” motion with the cube; **(C)** The hand performs a pinch to power transition against gravity, drawing the cube up from the support surface into a power grasp.

First, it was demonstrated that the hand could successfully perform the roll, yaw, and left-right shift primitives for the wooden cubes, knitted cube, foam cube, and rubber duck continuously in both directions with gravity pointing downwards into the palm (Figure 7). These motions were performed on loop and video of the task was recorded. Next, the hand was set up to perform a power to pinch manipulative motion of the red ball repeatedly, and video was taken of the task (Figure 8). Next, these tasks were repeated with the painted wooden cube with the hand flipped upside down, so that gravity pointed away from the palm. An additional motion was programmed into the controller that would pick up the cube from a surface below the hand, essentially completing a pinch to power primitive against gravity (Figure 8).

**FIGURE 9:**
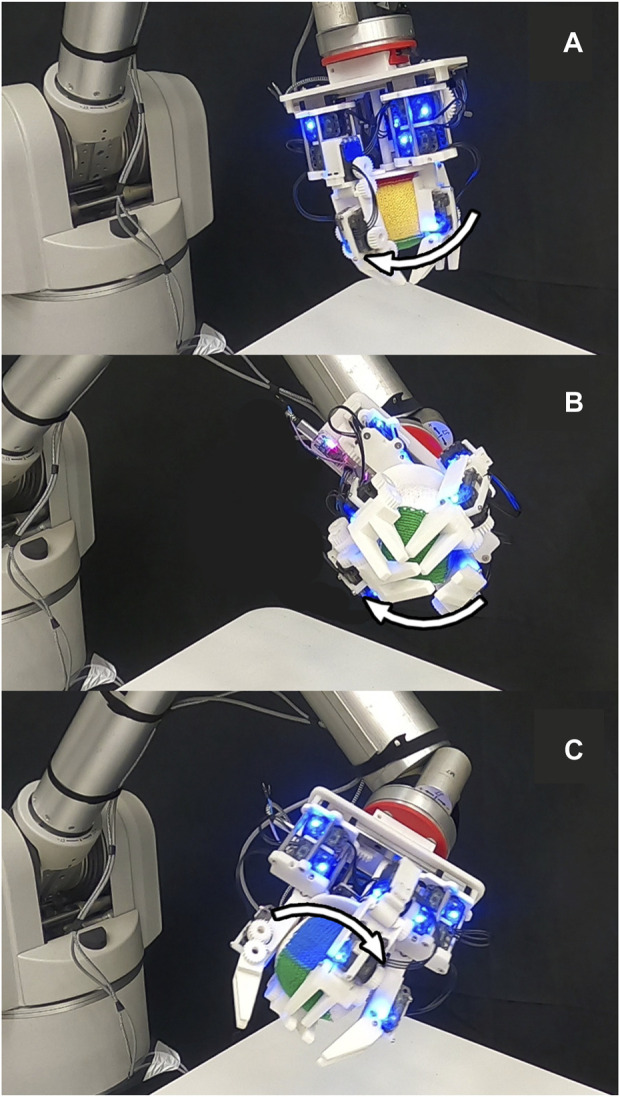
The Model B continuously reoriented the knitted cube while the WAM moved the hand through space from position **(A)**, to **(B)**, to **(C)**-while constantly changing the hand’s orientation with respect to gravity.

**FIGURE 10:**
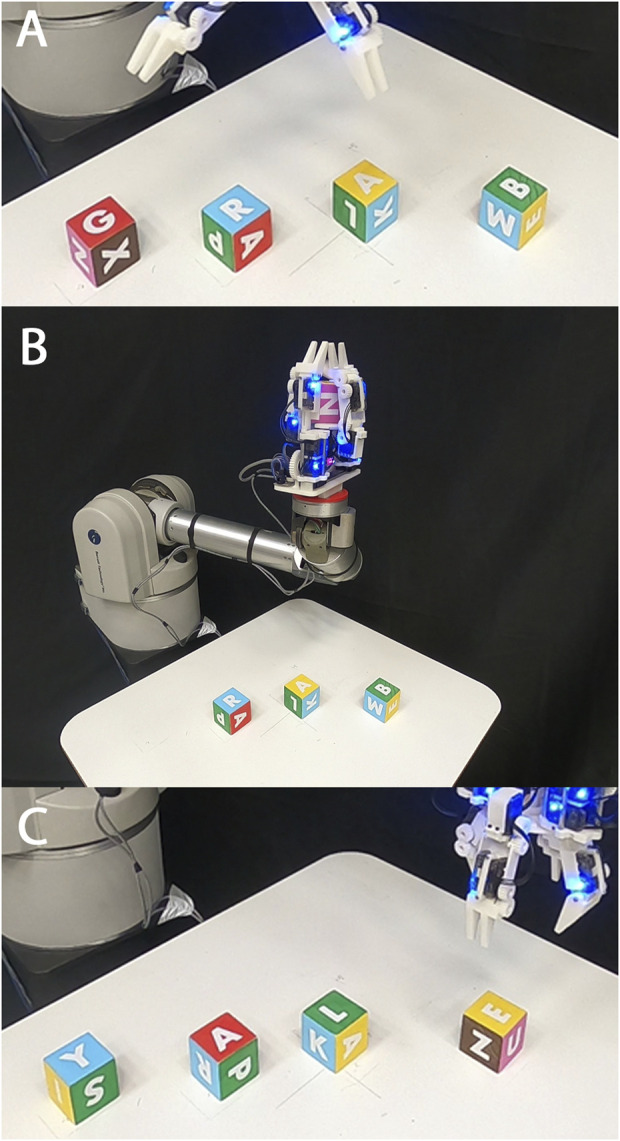
The Model B reoriented four painted cubes from G-R-A-B [panel **(A)**] to Y-A-L-E [panel **(C)**], transitioning the hand from an overhead configuration **(A)** to a vertical configuration **(B)** before performing any manipulation primitives.

**FIGURE 11:**
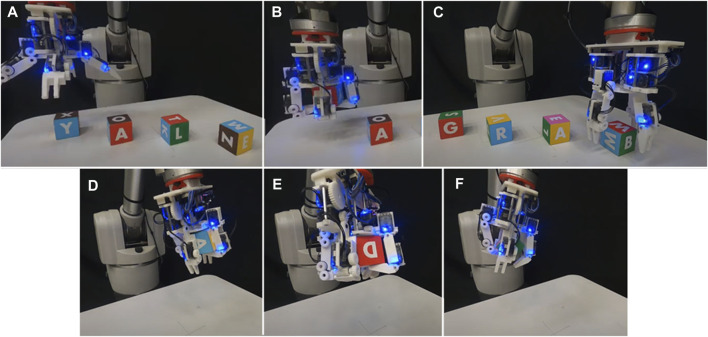
The Model B performed manipulation tasks with gravity pointing away from the palm. **(A)** The hand begins to reorient four painted wooden cubes from Y-A-L-E to G-R-A-B; **(B)** The hand performs a sequence of pre-determined open-loop manipulation primitives on the first cube, transitioning its front-facing side from the letter Y to the letter G; **(C)** The hand completes the task of reorienting all cubes; **(D)** The hand performs a “yaw” motion to the cube while moving through space; **(E)** The hand performs a “roll” motion to the cube while moving through space; **(F)** the hand performs a “yaw” motion.

After performing bench top tasks, the hand was fitted to the WAM arm and made to grasp a wide array of objects. These objects included a painted wooden block, a plastic orange, stackable plastic cups, a rubber duck, a 3D printed Stanford bunny, a plastic toy car, a golf ball, and a diceThese tasks were performed to show the range of object sizes that can easily be accommodated by the hand. Next, the hand was commanded to perform the roll task on the knitted cube, as the arm continuously moved the hand through different configurations in space (Figure 9). This task demonstrates that the manipulation of some objects is not dependent on a fixed gravity vector. Next, the hand was made to continually manipulate the painted wooden cube using the yaw and roll motions while the hand’s configuration was continuously moved through space. This was possible because of the caging grasps that were maintained during all manipulation primitives, allowing the hand to move an object but not drop it. Finally, the hand was programmed to grasp and manipulate four painted blocks, changing their upward facing letters from G-R-A-B to Y-A-L-E, chaining together manipulation primitives and grasps to accomplish a real world task. First, this task was performed with gravity facing downwards into the palm (the hand facing upwards) (Figure 10). Last, this task was completed again with the hand upside down, enabling the task to be completed much faster without a large motion of the arm to reconfigure the hand (Figure 11). Video was recorded of all grasps and WAM manipulation demos and can be seen in the Supplementary Video S1.

## 6 Discussion

This paper presents the first ever formulation of the potential energy-based forward motion model to three dimensions. We demonstrate how that can be used to compare the theoretical spatial manipulation performance of hand designs, and use this comparison to design a new hand, the Model B. The physical open loop manipulation capabilities of this hand were demonstrated and are shown to be capable of robustly manipulating a variety of objects.

There are many strong assumptions that went into this work that are worthy of discussion. Namely, the potential energy based forward motion model does not take friction into account. To date, we have not investigated the role that friction plays in the utility of this model. Also, at best we have only created a discrete representation of the actuator capabilities of each hand, meaning that a hand’s true continuous performance will never be fully captured using the methods in this paper. A large reason for this is that the more densely actuation input is sampled, the more computationally prohibitive the problem becomes. Despite these assumptions, we believe that in many cases the energy model is a useful tool and does a good job of approximating the motion of an object (see (Bircher et al., 2021) for data on this). To that end, we believe that it would be particularly useful as a motion model in a real time closed loop controller for manipulation, though that must be saved for future work. While our previous work (Bircher et al., 2021) designed hands specifically for good manipulation while caging, this work did not quantify the caging ability of hands. The reason for this is that it is much more challenging and computationally expensive to implement a metric for spatial caging than the planar caging technique used in our previous work. Rather than try to quantify a hand’s caging abilities, we instead chose to use our intuition to design a hand that could reasonably cage objects within a certain size range. Indeed, looking at the results of our experimental work, it is clear that the Model B is successful at caging objects of a certain size, as it can perform manipulation primitives while changing the configuration of the hand with respect to the gravity vector without object ejection.

## Data Availability

The original contributions presented in the study are included in the article/Supplementary Material, further inquiries can be directed to the corresponding author.
